# Comparative health technology assessment of robotic-assisted, direct manual laparoscopic and open surgery: a prospective study

**DOI:** 10.1007/s00464-016-4991-x

**Published:** 2016-06-17

**Authors:** Giuseppe Turchetti, Francesca Pierotti, Ilaria Palla, Stefania Manetti, Cinzia Freschi, Vincenzo Ferrari, Alfred Cuschieri

**Affiliations:** 1 Scuola Superiore Sant’Anna, Piazza Martiri della Libertà, 33, 56127 Pisa, Italy; 2 EndoCAS Center, University of Pisa, Pisa, Italy; 3 Institute for Medical Science and Technology, University of Dundee, Dundee, Scotland, UK

**Keywords:** Health technology assessment, Robotic surgery, da Vinci, Economic evaluation in health care, Economics of innovation

## Abstract

**Background:**

Despite many publications reporting on the increased hospital cost of robotic-assisted surgery (RAS) compared to direct manual laparoscopic surgery (DMLS) and open surgery (OS), the reported health economic studies lack details on clinical outcome, precluding valid health technology assessment (HTA).

**Methods:**

The present prospective study reports total cost analysis on 699 patients undergoing general surgical, gynecological and thoracic operations between 2011 and 2014 in the Italian Public Health Service, during which period eight major teaching hospitals treated the patients. The study compared total healthcare costs of RAS, DMLS and OS based on prospectively collected data on patient outcome in addition to healthcare costs incurred by the three approaches.

**Results:**

The cost of RAS operations was significantly higher than that of OS and DMLS for both gynecological and thoracic operations (*p* < 0.001). The study showed no significant difference in total costs between OS and DMLS. Total costs of general surgery RAS were significantly higher than those of OS (*p* < 0.001), but not against DMLS general surgery. Indirect costs were significantly lower in RAS compared to both DMLS general surgery and OS gynecological surgery due to the shorter length of hospital stay of RAS approach (*p* < 0.001). Additionally, in all specialties compared to OS, patients treated by RAS experienced a quicker recovery and significantly less pain during the hospitalization and after discharge.

**Conclusions:**

The present HTA while confirming higher total healthcare costs for RAS operations identified significant clinical benefits which may justify the increased expenditure incurred by this approach.

**Electronic supplementary material:**

The online version of this article (doi:10.1007/s00464-016-4991-x) contains supplementary material, which is available to authorized users.

Since 2000, the da Vinci surgical robot (Intuitive Surgical) has been the only system used clinically as an alternative to DMLS for the vast majority of laparoscopic operations. In 2013, surgeons performed approximately 523,000 robotic procedures worldwide, the commonest being hysterectomy and prostatectomy [1]. RAS has several advantages over DMLS, as the wristed end effectors permit seven degrees of freedom, thereby overcoming the kinematic constraints of DMLS, which together with motion scaling and HD stereoscopic imaging facilitates the execution of laparoscopic operations. One of the barriers to widespread adoption of this technology is its high capital, running and maintenance costs [2]. Health technology assessment (HTA) is a multidisciplinary evaluation of the clinical, economical, organizational and ethical implications concerning the adoption of new technologies, designed to provide healthcare providers with the relevant information necessary for informed decisions [3–6]. Although ideally performed when new and expensive technologies are first introduced, such an HTA has not been reported.

## Materials and methods

### Study design

The study enrolled patients from four regions (Lombardy, Piedmont, Tuscany and Lazio) by eight Italian major academic teaching hospitals during the period from February 2011 to May 2014 (details in appendix). The study as designed fulfilled all the required criteria of a HTA comparative study: prospectively collected non-randomized data on all consecutively enrolled patients, detailed analysis of costs of treatment from admission to 1 week after discharge, and evidence that clinical outcome was not demonstrably jeopardized by any of the three approaches used. The decision on the surgical approach was made by the attending physician in consultation with the patient. A case study form designed specifically for the collection of both clinical and health economic data was used. It comprised the following sections: enrollment/admission (*T*_0_); first follow-up (*T*_1_) 1 month after discharge; subsequent follow-ups (*T*_3_, *T*_4_, *T*_6_) at 3, 4, 6 months. An additional form was used for data from patients needing re-intervention during hospitalization or re-admission within 30 days of discharge.

A Web-based ad hoc database was developed for data collection using EasyPHP to create dynamic Web pages for data access and analysis. Patients’ confidentiality was by data anonymization using an alphanumeric univocal code. Each participating hospital identified a data manager, who accessed the data collection platform by username and password. Knowledge Discovery in Data process was implemented by different software and programming languages for automation of the data collection, extraction and analysis.

### Clinical assessment

The *T*_0_ stage included admission, operation, and postoperative course, including any postoperative re-intervention. It collected data on:

1. operating time (min) defined as interval between entry and exit of patient from the operative room (OR);
2. length of stay (days) including any re-admissions;
3. pain level diary by visual linear analogue scale from admission until 7 days post-discharge;
4. conversions classified as ‘enforced’ and ‘elective,’ using accepted definitions;
5. morbidity;
6. deaths including those following re-admission.

### Assessment of health costs

Direct healthcare costs were obtained by interviews conducted with an official from the Accounting Department of the hospitals involved in the study. It included the hourly cost of all staff working in the OR, daily cost of stay in wards and intensive care units; purchase cost of disposables and devices; and retail price per unit dose of drugs used [7]. Costs of laboratory tests, instrumental investigations and specialist visits were based on National Tariffs List of Outpatient Specialist Care of the four regions [8–11].

Direct non-healthcare costs were estimated using the replacement value [12] and included data on patient’s and care provider’s expenditure on food, accommodation and transport. Indirect costs based on loss of productivity were calculated using the human capital approach [13]. Specifically, productivity losses were estimated from patient’s hospital stay and expected income and employment. Currency conversions from Euros to US dollars (€1 = $1.112) were calculated as of May 21, 2015 (http://www.oanda.com/lang/it).

### Statistical analysis

Pearson Chi-square and Fisher’s exact tests were used to compare frequencies among groups. Continuous variables were analyzed by analysis of variance, or by nonparametric Kruskal–Wallis test depending on distribution of the data. Bonferroni post hoc tests or Mann–Whitney test with Bonferroni adjustment of *p* value was used for post hoc comparisons. Mixed-effects ML regression models for repeated measures were used to evaluate the level of pain during hospitalization and at home. Separated models were performed for each specialty. Variables with *p* value <0.001 on univariate analysis were included in the multivariate analysis. Statistical significance was set at 5 %.

## Results

The study recruited 699 patients who underwent operations in general surgery (*n* = 310), gynecology (*n* = 175) and thoracic surgery (*n* = 214) (Table 1).

**Table 1:** Patients enrolled per center, surgical technique and type of intervention

Surgical specialty	Type of intervention	RAS	DMLS	OS	Total
*University Hospital of Pisa*					
Thoracic surgery	Pulmonary lobectomy	36	0	22	58
	Thymectomy	9	0	8	17
Gynecological surgery	Hysterectomy for benign disease	34	18	18	70
	Hysterectomy for carcinoma	21	1	2	24
	Radical hysterectomy	8	0	6	14
	Myomectomy	31	12	23	66
	Removal of uterus	1	0	0	1
	Pelvis endometriosis	1	0	0	1
General surgery	Pancreatectomy	1	0	0	1
	Radical prostatectomy	17	0	17	34
	Cholecystectomy	0	1	0	1
	Hemicolectomy	0	0	1	1
	Anterior resection of rectum	0	1	1	2
	Adrenalectomy	2	0	0	2
Total per Center		162	33	98	293
*Hospital of Alessandria*					
General surgery	Anterior resection of rectum	32	0	0	32
*Hospital of Arezzo*					
General surgery	Anterior resection of rectum	12	9	3	24
*Campus Biomedico of Roma*					
General surgery	Anterior resection of rectum	11	9	0	20
	Abdomino-perineal resection	0	1	0	1
	Hemicolectomy	3	2	1	6
Total per Center		14	12	1	27
*Hospital of Grosseto*					
General surgery	Anterior resection of rectum	13	12	4	29
*European Oncology Institute of Milano*					
Thoracic surgery	Pulmonary lobectomy	20	34	46	100
	Thymectomy	0	0	6	6
	Pneumonectomy	2	0	2	4
	Segmentectomy	8	2	16	26
	Wedge	0	2	0	2
	Chest wall	1	0	0	1
Total per Center		31	38	70	139
*Le Molinette University Hospital of Torino*					
General surgery	Anterior resection of rectum	0	45	1	46
	Gastric bypass	56	33	1	80
	Radical prostatectomy	0	0	7	7
Total per Center		56	78	9	143
*San Matteo University Hospital of Pavia*					
General surgery	Cholecystectomy	0	0	12	12
Total		332	182	185	699

### Socio-demographic details

Of the 310 patients who underwent general surgical operations, 161 (52 %) underwent RAS, 113 (36 %) DMLS and 36 (12 %) OS. The characteristics of the groups differed. Thus, patients treated by OS were more likely to be male (*p* < 0.001). Additionally, patients undergoing RAS were significantly younger (60 ± 15 years) with respect to DMLS (65 ± 14 years) and OS (72 ± 8 years) (*p* < 0.001). The employment status of the patients also differed (*p* = 0.002): the majority of patients treated by OS were retired (78 %), while 37 % of the patients who underwent RAS were employed.

The approach in patients undergoing gynecological operations was 95 (54 %) by RAS, 31 (18 %) by DMLS and 49 (28 %) by OS. The mean age varied significantly (*p* = 0.01), with those treated by OS being significantly younger (47 ± 11 years) than those operated by RAS (54 ± 13 years).

The approach in patients requiring thoracic surgery was 100 (47 %) by OS, 76 (36 %) by RAS and 38 (18 %) by DMLS. No significant differences among the groups were found in the gender distribution, but the mean age was significantly lower (*p* = 0.01) in the RAS group, 64 ± 11 years, compared to both the DMLS group (69 ± 7 years) and the OS group (67 ± 11). The employment status was different (*p* = 0.008) with the highest proportion of retired patients being in the DMLS (74 %), whereas patients treated by RAS were more likely to be employed (41 %).

### Clinical results and outcome

In general surgery, the hospital stay was significantly shorter in the RAS compared with DMLS and OS (*p* < 0.001). In gynecological surgery, the hospital stay was significantly shorter in the RAS versus OS (*p* < 0.001), but not against DMLS. Likewise, the hospital stay after thoracic surgery was significantly shorter in RAS compared to the OS group (*p* < 0.001), but not against DMLS. Additionally, in both gynecological and thoracic surgery, the hospital stay was significantly shorter after DMLS compared with OS (*p* < 0.001). The operating time was significantly longer in both RAS general and thoracic surgery compared to OS and DMLS (*p* < 0.001 and *p* = 0.03, respectively). In gynecological surgery, there were no significant differences in operating times between the groups (Table 2).

**Table 2:** Clinical outcome: length of hospital stay and operating time

Specialty/technique	Median [25–75 %]	*p* value RAS versus DMLS	*p* value RAS versus OS
*Length of stay (days)*			
General (*n* = 310)			
RAS (*n* = 161)	6.0 [5.0–8.0]	*p* < 0.001	*p* < 0.001
DMLS (*n* = 113)	8.0 [6.00–12.0]		
OS (*n* = 36)	8.5 [7.00–10.0]		
Gynecological (*n* = 175)			
RAS (*n* = 95)	3.0 [2.0–3.0]	*p* = 0.17	*p* < 0.001
DMLS (*n* = 31)	3.0 [3.0–4.0]		
OS (*n* = 49)	4.0 [4.0–6.0]		
Thoracic (*n* = 214)			
RAS (*n* = 76)	6.0 [5.0–7.0]	*p* = 0.87	*p* < 0.001
DMLS (*n* = 38)	6.0 [5.0–7.0]		
OS (*n* = 100)	7.0 [6.0–9.0]		
*Operating time (min)*			
General			
RAS	380.0 [335.0–430.0]	*p* < 0.001	*p* < 0.001
DMLS	285.0 [240.0-345.0]		
OS	257.5 [225.0–300.0]		
Gynecological			
RAS	210.0 [170.0–260.00]	*p* = 0.52	*p* = 0.11
DMLS	180.0 [145.0–225.0]		
OS	185.0 [145.0–230.0]		
Thoracic			
RAS	299.5 [248.5–359.5]	*p* = 0.03	*p* < 0.001
DMLS	266.0 [232.0–310.0]		
OS	224.5 [181.0–261.0]		

### Conversions, re-interventions and re-admissions

Total conversions during *T*_0_ phase were 22: 10 in RAS and 12 in DMLS operations. In general surgery, the 18 conversions consisted of 6 in RAS (3 to DMLS, 3 to OS) and 12 in DMLS (12 to OS—10 elective and 2 enforced). In gynecology, there was one elective conversion from RAS to DMLS. In thoracic surgery, two out of three conversions to OS were enforced and the third, elective. One elective conversion occurred during a re-intervention in *T*_0_ in RAS general surgery to OS (see Appendix Table A1).

Nine patients required re-intervention during *T*_0_ phase: 5 in DMLS general surgery, 2 after RAS. Two other patients required re-intervention in thoracic surgery: 1 after OS and another after RAS (see Appendix Table A2).

Eight patients required re-admissions within 30 days of discharge from hospital: 4 in general surgery, of which 1 after RAS, 2 after DMLS and 1 after OS. The two re-admissions in gynecology occurred after DMLS and OS. In thoracic surgery, two patients were re-admitted: 1 each after RAS and OS. These differences between the three approaches were not significant (see Appendix Table A3).

### Intraoperative complications

These were encountered during 16 operations, being minor in 6 and major in 10. Minor complications occurred in general (1 DMLS and 2 RAS) and in thoracic surgery (all 3 OS). None altered the surgical treatment or subsequent clinical course. Major complications were encountered in all three specialties: 1 during RAS general surgery, 5 during gynecological operations (2 RAS and 3 OS) and 4 thoracic (2 RAS and 2 OS), again without significant differences between the groups (see Appendix Table A4).

### Postoperative morbidity

Total postoperative morbidity comprised 17 minor and 35 major complications. The former were largely encountered in general surgery (4 after DMLS, 3 after OS and 6 after RAS), 1 after gynecological DMLS and 3 after open thoracic surgery. Major complications were encountered in general surgery (*n* = 19) and thoracic surgery (*n* = 16). The major complications after general surgery operations were encountered in 10 after RAS, 7 after DMLS and 2 after OS. The distribution of major complications after thoracic operations (9 after RAS, 6 after OS and 1 after DMLS) was similar (see Appendix Table A5).

There were 54 medical postoperative complications: 22 in general surgery (9 in RAS, 8 after DMLS and 5 after OS), 1 in gynecology after DMLS and 31 after thoracic surgery (18 after OS, 11 in RAS and 2 after DMLS). The incidence of medical complications was similar between the groups (see Appendix Table A6).

### Clinical benefits documented by present study

The most significant benefit of RAS operations across the three specialties was the reduced pain after surgery compared to OS and DMLS (Table 3).

**Table 3:** Pain level: mixed-effects ML regression models for repeated measures

	Coeff.	95 % CI	*p* value
*Pain during hospitalization*			
General surgery			
Unadjusted			
RAS	Ref.	–	–
DMLS	0.023	−0.105 to 0.150	0.726
OS	0.243	0.035–0.451	0.022
Adjusted^a^			
RAS	Ref.	–	–
DMLS	−0.036	−0.161 to 0.088	0.564
OS	0.227	0.027–0.427	0.026
Gynecological surgery			
Unadjusted			
RAS	Ref.	–	–
DMLS	0.331	0.572–0.605	0.018
OS	0.518	0.295–0.740	<0.001
Adjusted^b^			
RAS	Ref.	–	–
DMLS	0.299	0.026–0.572	0.032
OS	0.428	0.187–0.668	<0.001
Thoracic surgery			
Unadjusted			
RAS	Ref.	–	–
DMLS	0.417	0.188–0.647	<0.001
OS	−0.005	−0.185 to 0.175	0.957
Adjusted^c^			
RAS	Ref.	–	–
DMLS	0.312	0.114–0.511	0.002
OS	−0.023	−0.178 to 0.132	0.768
*Pain after discharge*			
General surgery			
Unadjusted			
RAS	Ref.	–	–
DMLS	0.004	−0.095 to 0.102	0.943
OS	0.343	0.178–0.508	<0.001
Adjusted^d^			
RAS	Ref.	–	–
DMLS	−0.046	−0.141 to 0.049	0.341
OS	0.255	0.098–0.412	0.001
Gynecological surgery			
Unadjusted			
RAS	Ref.	–	–
DMLS	0.333	0.128–0.537	0.001
OS	0.539	0.365–0.713	<0.001
Adjusted^e^			
RAS	Ref.	–	–
DMLS	0.221	0.025–0.417	0.027
OS	0.275	0.110–0.440	0.001
Thoracic surgery			
Unadjusted			
RAS	Ref.	–	–
DMLS	0.352	0.134–0.570	0.002
OS	0.104	−0.065 to 0.273	0.229
Adjusted^f^			
RAS	Ref.	–	–
DMLS	0.259	0.070–0.448	0.007
OS	0.104	−0.042 to 0.250	0.164

^a^Adjusted model for length of stay and conversions

^b^Adjusted model for length of stay

^c^Adjusted model for pain at home

^d^Adjusted model for pain during hospitalization and conversions

^e^Adjusted model for pain during hospitalization and age

^f^Adjusted model for pain during hospitalization, postoperative complications and re-interventions

### Pain during hospitalization

In general surgery, patients treated by RAS experienced less pain compared to OS (*p* = 0.026), with the pain level being similar to that experienced by DMLS patients. On adjusting for length of stay, the pain level was significantly lower in gynecological RAS versus both DMLS (*p* = 0.032) and OS (*p* < 0.001). On adjusting for pain after discharge, the pain level was significantly lower in thoracic RAS compared to DMLS (*p* = 0.002), but not against OS.

### Pain after discharge

The pain level after discharge was significantly lower in general RAS patients compared to OS (*p* = 0.001), but not against DMLS. After gynecological RAS, patients experienced less pain compared to both DMLS (*p* = 0.027) and OS (*p* = 0.001). After thoracic RAS, the pain level was significantly lower compared to DMLS (*p* = 0.007), but not with patients after OS.

Almost all patients undergoing RAS and DMLS in general and gynecological surgery reported their ability for daily activities and exercises to be good, very good or excellent, significantly better than after OS (*p* = 0.001 and *p* < 0.001, respectively). Hence, their quality of life was better during this post-discharge period.

### Cost analysis

In the gynecological and thoracic specialties, the RAS approach incurred significantly higher direct healthcare costs compared to both DMLS and OS (*p* < 0.001), while costs between OS and DMLS were similar. In general surgery, direct healthcare costs of RAS were higher than those of OS (*p* < 0.001) but similar to those incurred by DMLS. General surgery performed by the open approach incurred higher direct healthcare costs compared to DMLS operations (*p* < 0.001).

In general surgery, direct non-healthcare costs were similar between the three approaches, whereas in gynecology, the RAS approach incurred significantly higher costs compared to DMLS (*p* = 0.01). In thoracic surgery, direct non-healthcare costs were higher for DMLS compared to RAS (*p* = 0.003) and OS (*p* = 0.006).

The only significant differences in indirect costs were observed in general and gynecological surgery. In general surgery, RAS indirect costs were lower than those of DMLS (*p* < 0.05), whereas in gynecology, RAS indirect costs were lower than those of OS (*p* < 0.001).

Total costs of RAS were significantly higher than those of the two other approaches for gynecological and thoracic specialties (*p* < 0.001), but total costs for both OS and DMLS operations were similar. In general surgery, total costs of RAS were higher compared to OS (*p* < 0.001), but not against DMLS. Full details of the cost analysis data are shown in Table 4. After adjusting for centers and/or patients characteristics for both direct healthcare costs and overall costs, the RAS approach incurred significantly higher costs (Table 5).

**Table 4:** Costs associated with the different surgical approaches by specialty

Technique	Median [25–75 %]		*p* value	
	*€*	*$*	RAS versus DMLS	RAS versus OS
*General surgery (n* *=* *310)*				
Total direct healthcare costs				
RAS	9928 [9158–10,893]	11,038 [10,181–12,110]	*p* = 0.52	*p* < 0.001
DMLS	9997 [7322–11,095]	11,114 [8140–12,335]		
OS	6764 [6084–8131]	7520 [6764–9040]		
Total direct non-healthcare costs				
RAS	585 [340–922]	650 [378–1025]	*p* = 0.94	*p* = 0.44
DMLS	564 [399–878]	627 [443–976]		
OS	516 [368–889]	574 [409–988]		
Indirect costs				
RAS	1064 [649–1313]	1183 [721–1460]	*p* = 0.02	*p* = 0.28
DMLS	1313 [1021–1525]	1460 [1135–1695]		
OS	1275 [1034–1543]	1417 [1150–1715]		
Total costs				
RAS	10,822 [9995–12,065]	12,031 [11,112–13,413]	*p* = 0.35	*p* < 0.001
DMLS	10,778 [8660–12,242]	11,983 [9628–13,610]		
OS	7267 [6613–8684]	8079 [7352–9655]		
*Gynecological surgery (n* *=* *175)*				
Total direct healthcare costs				
RAS	7902 [7507–8499]	8785 [8346–9449]	*p* < 0.001	*p* < 0.001
DMLS	4231 [3878–5129]	4704 [4311–5702]		
OS	4328 [3768–5610]	4812 [4189–6237]		
Total direct non-healthcare costs				
RAS	351 [281–523]	390 [312–581]	*p* = 0.01	*p* = 0.76
DMLS	281 [210–381]	312 [233–423]		
OS	341 [260–590]	379 [289–656]		
Indirect costs				
RAS	683 [502–859]	759 [558–955]	*p* = 0.38	*p* < 0.001
DMLS	739 [515–859]	821 [572–955]		
OS	964 [749–1202]	1072 [833–1336]		
Total costs				
RAS	8739 [8110–9757]	9716 [9016–10,847]	*p* < 0.001	*p* < 0.001
DMLS	4936 [4733–6249]	5488 [5262–6947]		
OS	5753 [4609–8378]	6396 [5124–9314]		
*Thoracic surgery* *(n* *=* *214)*				
Total direct healthcare costs				
RAS	11,917 [10,676–13,095]	13,249 [11,869–14,558]	*p* < 0.001	*p* < 0.001
DMLS	8887 [7738–9839]	9880 [8603–10,939]		
OS	8884 [7824–9878]	9877 [8698–10,982]		
Total direct non-healthcare costs				
RAS	987 [595–1450]	1097 [661–1612]	*p* = 0.003	*p* = 0.32
DMLS	2065 [801–3655]	2296 [890–4063]		
OS	1043 [702–1626]	1160 [780–1808]		
Indirect costs				
RAS	1202 [1053–2363]	1336 [1171–2627]	*p* = 0.28	*p* = 1.00
DMLS	1153 [886–1520]	1282 [985–1690]		
OS	1342 [1114–1564]	1492 [1239–1739]		
Total costs				
RAS	13,856 [12,343–15,291]	15,405 [13,722–17,000]	*p* < 0.001	*p* < 0.001
DMLS	10,888 [9178–13,357]	12,105 [10,204–14,850]		
OS	10,574 [9188–11,737]	11,756 [10,215–13,049]

**Table 5:** Adjusted costs differences by specialty

Specialty/technique	Coeff.	SE	*p* value	Inf. 95 %	Sup. 95 %
*Total direct healthcare costs*					
General^a^					
DMLS versus RAS	−1256.472	424.2759	0.003	−2088.037	−424.9067
OS versus RAS	−3242.441	458.0922	<0.001	−4140.285	−2344.596
Gynecological^b^					
DMLS versus RAS	−3256.859	343.91	<0.001	−3930.91	−2582.808
OS versus RAS	−2609.776	325.5717	<0.001	−3247.885	−1971.667
Thoracic^a^					
DMLS versus RAS	−3883.981	534.3085	<0.001	−4931.206	−2836.755
OS versus RAS	−3566.99	409.8714	<0.001	−4370.323	−2763.657
*Total costs*					
General^a^					
DMLS versus RAS	−1299.863	466.9079	0.005	−2214.986	−384.7402
OS versus RAS	−3542.22	508.8817	<0.001	−4539.61	−2544.83
Gynecological^b^					
DMLS versus RAS	−3380.546	421.8935	<0.001	−4207.442	−2553.65
OS versus RAS	−2248.316	411.214	<0.001	−3054.28	−1442.351
Thoracic^a^					
DMLS versus RAS	−3775.765	676.8711	<0.001	−5102.408	−2449.122
OS versus RAS	−4001.056	499.1097	<0.001	−4979.294	−3022.819

^a^Adjusted model considering centers’ effects

^b^Adjusted model for age

## Discussion

As required by HTA, the present prospective comparative study collected data on clinical outcome in addition to health economic costs. The study was necessary as the HTA question: *does RAS represent good value for money?* has not been answered; especially as from the health providers’ and societal perspectives, the issue is not simply that RAS is more expensive, but rather—*is the extra cost of RAS justified by improved patient outcome?* The present study answers, to a limited extent, the second question. Thus, while confirming higher direct healthcare costs (but not direct non-medical and indirect health costs), it documents that RAS reduces hospital stay and pain before and after discharge. The pain reported by patients after discharge associated with essential daily activities during the first week enhances the quality of life during this period. An additional benefit of RAS is reduced hospital stay with a trend toward accelerated recovery leading to less pain and improved quality of life during the first post-discharge week, ranging from good to excellent.

The alleged benefit of RAS is based on retrospective studies and mixed systemic reviews/meta-analysis. It is not surprising that retrospective studies often produce conflicting results, due to the influence of uncontrolled variables. This is exemplified by distal pancreatectomy. In one study which compared DMLS with RAS for distal pancreatectomy for tumors, spleen-preserving RAS was associated with significantly higher spleen preservation rates, shorter operating time, less blood loss and shorter mean hospital stay [14]. However, a similar but prospective non-randomized study did not report any significant difference. Depending on availability of robot, all patients suitable for distal pancreatectomy were assigned either to DMLS or to RAS DP. The median operative time was longer, and procedures cost was double in RAS group. Conversion to open and the median length of postoperative hospital stay were similar, as was pancreatic fistula rate (57 and 50 %) [15]. In colorectal surgery, a meta-analysis on RAS total mesorectal resection for rectal cancer compared DMLS-TME with RAS-TME. The latter exhibited significantly fewer conversions, lower positive circumferential resection margins, and erectile dysfunction [16]. Thus for this operation, RAS appears to carry clinical benefit over DMLS, despite increased cost.

Two publications in gynecology on health-related quality of life [17, 18] reported results in favor of RAS, which are in agreement with the results of the present prospective HTA study. The first [17] used a HRQoL questionnaire to study patient satisfaction in patients undergoing RAS hysterectomy for cancer. The HRQoL questionnaire was completed at the first postoperative visit in 109 patients. These reported the pain level as being highest on the second postoperative day, but two-thirds reported no pain by the first postoperative visit, and only 18 % of patients needed narcotics for pain control. Most patients resumed normal activities within 11 days after surgery and reported a satisfaction rating of 6.7 on a 7-point scale. The other report [18] studied the HRQoL in 211 patients also undergoing RAS resection of gynecologic cancer. The patients completed a QoL questionnaire before surgery and postoperatively at 1 and 3 weeks, and at 3, 6 and 12 months. Overall HRQoL and body image decreased at 1 week after surgery but returned to baseline by 3 weeks. Physical and functional well-being decreased at 1 week after surgery but returned to baseline by 3 months. Another study [19] compared the postoperative pain management and costs in endometrial cancer patients who underwent a RAS hysterectomy. In this study, RAS patients needed a lower number of drug interventions (*p* < 0.001), with a 50 % reduction in the pain medication costs on the day of surgery (*p* < 0.01), and a 56 % cost reduction for the rest of their hospital stay (*p* < 0.01). The pain reduction demonstrated by this retrospective study is confirmed by the present prospective HTA study. A large statewide health economic study involving 2247 patients analyzed the utilization and hospital charges associated with RAS versus DMLS and OS treatment of endometrial cancer [20]. In this study, 29 % of patients were treated by RAS, 10 % by DMLS and 61 % by OS. The mean length of hospital stay was significantly shorter after RAS and DMLS compared to OS (*p* < 0.001). The median hospital charge was $51,569, $37,202 and $36,492, for RS, LS and OS (*p* < 0.001). A recent report in thoracic surgery is relevant to the present HTA study. The study was designed to determine a realistic medical fee for RAS thoracic surgery for the Japanese National Health Insurance System (JNHIS) introduced in 2012 [21]. It concluded that the projected cost to the JNHIS for RAS thoracic interventions would only be sustainable by institutions, which performed more than 300 RAS interventions per year.

In conclusion, the present HTA study indicates that the issue of increased costs of RAS is complex and multifactorial. RAS is likely to be cost beneficial in terms of reduced hospital stay, reduced pain and improved quality of life, provided certain conditions are met, including case load and case mix.

## Electronic supplementary material

Below is the link to the electronic supplementary material.

Supplementary material 1 (DOCX 74 kb)
