# Supplementary material for: Comparative health technology assessment of robotic-assisted, direct manual laparoscopic and open surgery: a prospective study
Source: Surg Endosc. 2016 Jun 17;31(2):543–51. doi: 10.1007/s00464-016-4991-x (PMC5266759; doi:10.1007/s00464-016-4991-x)
Supplement: Supplementary file 1 — Supplementary material 1 (DOCX 74 kb) [file 464_2016_4991_MOESM1_ESM.docx]

**PARTICIPATING HOSPITALS:**

University Hospital of Pisa, European Oncology Institute of Milano, Le Molinette University Hospital of Torino, San Matteo University Hospital of Pavia, Hospital of Alessandria, Hospital of Arezzo, Hospital of Grosseto and Campus Biomedico of Rome.

**HEALTH ECONOMICS – METHODOLOGY USED**

**Cost of surgical intervention**

Robotic intervention cost = robotic devices cost + cost operating room + operating time * (hourly cost of room nurse) + skin to skin time * [(number of surgeons * hourly cost of surgeon) + hourly cost of scrub nurse + hourly cost of professional or specialist nurse + hourly cost of unwashed nurse + hourly cost of assistant surgeon] + anesthesiologist time* [hourly cost of anesthesiologist + hourly cost of anesthesia technician] + setup-p-time * hourly cost of nurse + trocar time * hourly cost of surgeon

Not robotic intervention cost = same procedure replacing the robotic devices cost with not robotic devices cost. It is not considered setup and trocar time.

Number of surgeons in operating room: 3 in robotic and laparoscopic surgery; 4 in open approach

**Hospitalization cost**

Total cost estimated from cost/ day of hospitalization. Daily cost of hospitalization is constituted by:

-Cost of non-medical staff (nursing, administrative and technical)

-Amortization of equipment

-General costs (food, administrative expense etc.)

**Total direct healthcare costs**

= Total tariffs (tests and visits) + Drugs costs + Intra-operative costs + intensive care unit costs + hospitalization costs. We also consider eventual costs of re-interventions.

**Direct non-healthcare costs**

= caregiver expenditure (food, accommodation and transport) + patient expenditure (food, accommodation and transport) + informal care (informal assistance of caregiver outside working time).

**Indirect costs**

The indirect costs are estimated using the human capital approach. This method considers the working time lost by the patient (estimated on length of stay) and expected income according to work sector, role and work time (full time 8 hours, part time 4 hours, null 6 hours).

**Total costs T_0_**

= healthcare direct costs + non healthcare direct costs + indirect costs

**Total costs**

= total cost T0 + Total cost follow up T1 + Total cost next follow up+ Total cost re-admission

| *Surgical specialty* | *Type of intervention* | *Enforced conversion* | *Elective conversion* | *Total conversions* | *Total by type and technique* | *% conversion on total by type and technique* |
| --- | --- | --- | --- | --- | --- | --- |
| **University Hospital of Pisa** | | | | | | |
| *Thoracic surgery* | RAS thymectomy | 1 | 0 | 1 | 9 | 11.1 |
|  | RAS lobectomy | 1 | 1 | 2 | 36 | 5.5 |
| *Gynecological surgery* | RAS  myomectomy | 0 | 1 | 1 | 31 | 3.2 |
| **Hospital of Alessandria** | | | | | | |
| *General surgery* | RAS anterior resection of rectum | 1 | 1 | 2 | 32 | 6.2 |
| **Hospital of Arezzo** | | | | | | |
| *General surgery* | RAS anterior resection of rectum | 1 | 0 | 1 | 12 | 8.3 |
|  | DMLS anterior resection of rectum | 0 | 6 | 6 | 9 | 66.7 |
| **Campus Biomedico of Roma** | | | | | | |
| *General surgery* | RAS anterior resection of rectum | 0 | 1 | 1 | 11 | 9.1 |
|  | DMLS anterior resection of rectum | 0 | 1 | 1 | 9 | 11.1 |
| **Hospital of Grosseto** | | | | | | |
| *General surgery* | RAS anterior resection of rectum | 1 | 1* | 2 | 13 | 15.4 |
| **Le Molinette University Hospital of Torino** | | | | | | |
| *General surgery* | RAS bypass | 1 | 0 | 1 | 56 | 1.8 |
|  | DMLS anterior resection of rectum | 2 | 3 | 5 | 45 | 11.1 |
| **Total** | | **8** | **15** | **23** | **263** | **8.7** |

**Table A1.** *Conversions to open surgery*

*In re-intervention phase

**Table A2.** Re-interventions during T_0_ phase

| *Surgical specialty* | *Type of intervention* | *Number of re-interventions* | *Total by type and technique* | *% re-interventions on total by type and technique* |
| --- | --- | --- | --- | --- |
| **University Hospital of Pisa** | | | | |
| *Thoracic surgery* | OS thymectomy | 1 | 8 | 12.5 |
|  | RAS lobectomy | 1 | 36 | 2.8 |
| **Hospital of Arezzo** | | | | |
| *General surgery* | RAS anterior resection of rectum | 1 | 12 | 8.3 |
| **Hospital of Grosseto** | | | | |
| *General surgery* | RAS anterior resection of rectum | 1 | 13 | 7.7 |
|  | DMLS anterior resection of rectum | 1 | 12 | 8.3 |
| **Le Molinette University Hospital of Torino** | | | | |
| *General surgery* | DMLS bypass | 1 | 33 | 3.0 |
|  | DMLS anterior resection of rectum | 3 | 45 | 6.7 |
| **Total** | | **9** | **159** | **5.7** |

| *Surgical specialty* | *Type of intervention* | *Number of re-admissions* | *Total by type and technique* | *% re-admissions on total by type and technique* |
| --- | --- | --- | --- | --- |
| **University Hospital of Pisa** | | | | |
| *Gynecological surgery* | DMLS benign hysterectomy | 1 | 18 | 5.5 |
|  | OS myomectomy | 1 | 23 | 4.3 |
| *Thoracic*  *surgery* | OS thymectomy | 1 | 8 | 12.5 |
|  | RAS lobectomy | 1 | 36 | 2.8 |
| **Hospital of Alessandria** | | | | |
| *General*  *surgery* | RAS anterior resection of rectum | 1 | 32 | 3.1 |
| **Hospital of Grosseto** | | | | |
| *General*  *surgery* | DMLS anterior resection of rectum | 1 | 12 | 8.3 |
| **Le Molinette University Hospital of Pavia** | | | | |
| *General*  *surgery* | DMLS bypass | 1 | 33 | 9.1 |
|  | OS radical prostatectomy | 1 | 7 | 14.3 |
| **Total** |  | **8** | **169** | **4.8** |

**Table A3**. Re-admissions within 30 days of discharge from hospital

| *Surgical specialty* | *Type of intervention* | *Minor* | *Major* | *Total complications* | *Total by type and technique* | *% complications on total by type and technique* |
| --- | --- | --- | --- | --- | --- | --- |
| **University Hospital of Pisa** | | | | | | |
| *Thoracic surgery* | OS thymectomy | 1 | 0 | 1 | 8 | 12.5 |
|  | RAS lobectomy | 0 | 2 | 2 | 36 | 5.6 |
| *Gynecological*  *surgery* | OS hysterectomy for carcinoma | 0 | 1 | 1 | 2 | 50.0 |
|  | RAS  myomectomy | 0 | 1 | 1 | 31 | 3.2 |
|  | OS myomectomy | 0 | 2 | 2 | 23 | 8.7 |
|  | RAS radical hysterectomy | 0 | 1 | 1 | 8 | 12.5 |
| **Hospital of Alessandria** | | | | | | |
| *General*  *surgery* | RAS anterior resection of rectum | 1 | 0 | 1 | 32 | 3.1 |
| **Campus Biomedico of Roma** | | | | | | |
| *General*  *surgery* | RAS anterior resection of rectum | 0 | 1 | 1 | 11 | 9.1 |
| **European Oncology Institute of Milano** | | | | | | |
| *Thoracic surgery* | OS lobectomy | 2 | 0 | 2 | 46 | 4.3 |
|  | OS pneumonectomy | 0 | 1 | 1 | 2 | 50.0 |
|  | OS segmentectomy | 0 | 1 | 1 | 16 | 6.2 |
| **Le Molinette University Hospital of Torino** | | | | | | |
| *General*  *surgery* | DMLS anterior resection of rectum | 1 | 0 | 1 | 45 | 2.2 |
| **San Matteo University Hospital of Pavia** | | | | | | |
| *General*  *surgery* | RAS cholecystectomy | 1 | 0 | 1 | 12 | 8.3 |
| **Total** |  | **6** | **10** | **16** | **272** | **5.9** |

**Table A4.** Minor and major intra-operative complications

**Table A5.** Minor and major postoperative complications using standard surgical definitions

| *Surgical specialty* | *Type of intervention* | *Minor* | *Major* | *Tot*  *complications* | *Total by type and technique* | *% complications on total by type and technique* |
| --- | --- | --- | --- | --- | --- | --- |
| **University Hospital of Pisa** | | | | | | |
| *Thoracic surgery* | OS thymectomy | 1 | 0 | 1 | 8 | 12.5 |
|  | OS lobectomy | 1 | 3 | 4 | 22 | 18.2 |
|  | RAS lobectomy | 0 | 5 | 5 | 36 | 13.9 |
|  | RAS thymectomy | 0 | 1 | 1 | 9 | 11.1 |
| *Gynecological surgery* | DMLS myomectomy | 1 | 0 | 1 | 12 | 8.3 |
| *General surgery* | OS radical prostatectomy | 0 | 1 | 1 | 17 | 5.9 |
| **Hospital of Alessandria** | | | | | | |
| *General surgery* | RAS anterior resection of rectum | 2 | 4 | 6 | 32 | 18.7 |
| **Hospital of Arezzo** | | | | | | |
| *General surgery* | DMLS anterior resection of rectum | 2 | 0 | 2 | 9 | 22.2 |
|  | OS anterior resection of rectum | 1 | 0 | 1 | 3 | 33.3 |
|  | RAS anterior resection of rectum | 1 | 4 | 5 | 12 | 41.7 |
| **Campus Biomedico of Roma** | | | | | | |
| *General surgery* | DMLS anterior resection of rectum | 1 | 1 | 2 | 9 | 22.2 |
|  | OS hemicolectomy | 1 | 0 | 1 | 1 | 100.0 |
|  | RAS anterior resection of rectum | 3 | 1 | 4 | 11 | 36.4 |
| **Hospital of Grosseto** | | | | | | |
| *General surgery* | DMLS anterior resection of rectum | 0 | 1 | 1 | 12 | **8.3** |
|  | RAS anterior resection of rectum | 0 | 1 | 1 | 13 | **7.7** |
| **European Oncology Institute of Milano** | | | | | | |
| *Thoracic surgery* | OS pneumonectomy | 1 | 0 | 1 | 2 | 50.0 |
|  | DMLS lobectomy | 0 | 1 | 1 | 34 | 2.9 |
|  | OS lobectomy | 0 | 2 | 2 | 46 | 4.3 |
|  | OS thymectomy | 0 | 1 | 1 | 6 | 16.7 |
|  | RAS segmentectomy | 0 | 1 | 1 | 2 | 50.0 |
|  | RAS lobectomy | 0 | 2 | 2 | 20 | 10.0 |
| **Le Molinette University Hospital of Torino** | | | | | | |
| *General surgery* | DMLS anterior resection of rectum | 1 | 4 | 5 | 45 | 11.1 |
|  | OS radical prostatectomy | 1 | 1 | 2 | 7 | 28.6 |
|  | DMLS bypass | 0 | 1 | 1 | 33 | 3.0 |
| **Total** |  | **17** | **35** | **52** | **401** | **13.0** |

**Table A6.** Medical postoperative complications
